# The Emergence of the Omicron (B.1.1.529) SARS-CoV-2 Variant: What is the Impact on the Continued Pandemic?

**DOI:** 10.1007/s44197-022-00032-w

**Published:** 2022-01-28

**Authors:** Jaffar A. Al-Tawfiq, Van-Thuan Hoang, Nhat Le Bui, Dinh-Toi Chu, Ziad A. Memish

**Affiliations:** 1 Infectious Disease Unit, Specialty Internal Medicine, Johns Hopkins Aramco Healthcare, Dhahran, Saudi Arabia; 2 Department of Medicine, Indiana University School of Medicine, Indianapolis, IN USA; 3 Department of Medicine, Johns Hopkins University School of Medicine, Baltimore, MD USA; 4 Thai Binh University of Medicine and Pharmacy, Thai Binh, Vietnam; 5 Center for Biomedicine and Community Health, International School, Vietnam National University, Hanoi, Vietnam; 6 Director Research and Innovation Centre, King Saud Medical City, Ministry of Health and College of Medicine, Alfaisal University, Riyadh, Kingdom of Saudi Arabia; 7 Hubert Department of Global Health, Rollins School of Public Health, Emory University, Atlanta, GA USA

## Introduction

Since the emergence of the Coronavirus disease 2019 (COVID-19) in Dec 2019, the world continues to be facing a fierce pandemic characterized by multiple waves at various paces in different countries. Globally, a fourth wave had started in the last quarter of 2021. As of 19 December, over 273 million cases and over 5.3 million deaths have been reported globally [1]. Since the causative agent of Severe Acute Respiratory Syndrome Coronavirus 2 (SARS-CoV-2) is an RNA virus, the virus is subject to a significant number of mutations overtime. Most changes have little to no impact on the virus’ properties. However, some changes may affect the virus’s properties, such as how easily it spreads, the associated disease severity, or the performance of vaccines, therapeutic medicines, diagnostic tools, or other public health and social measures [2].. The Omicron variant was reported to world health organization (WHO) from South Africa (SA) on 24 November 2021 [3] and promptly classified as a variant of concern on 26 November 2021 [2] (Table 1). One of the most concerning features of the Omicron variant is the aggregation of 50 mutations, of which about 30 mutations were detected in the spike protein [4, 5]. These mutations are widely allocated at several proteins including NSP3, NSP4, NSP5, NSP6, NSP12, NSP14, S protein, envelope protein, membrane protein, and nucleocapsid protein [6]. However, the most feared mutations are those on the receptor-binding domain (RBD). The deletion in the S protein of (Δ69–70) is being used as a proxy for the detection of the omicron variant. This deletion gives a negative S gene but positive SARS-CoV-2 on the TaqPath PCR test [7].

**Table 1:** Most important SARS-CoV-2 variants and initial events

	Alpha	Beta	Gamma	Delta	Omicron
Pango lineage	B.1.1.7	B.1.351	P.1	B.1.617.2	B.1.1.529
Date of designation	18 December 2020	18 December 2020	11 January 2021	11 May 2021	26 November 2021
Initial Country	United Kingdom	South Africa	Brazil	India	South Africa, Hong Kong, Israel, Belgium
(Δ69–70) deletion as revealed by TaqPath PCR	Yes	No	No	No	Yes

The epidemiological situation in South Africa has been characterized by three distinct peaks in reported cases, the latest of which was predominantly the *Delta* variant. In recent weeks, SA experienced steep increase in community infections (concentrated in the Gauteng) dominated by the Omicron variant, fueling SA’s fourth wave of COVID-19. The first known confirmed infection with this variant was from a specimen collected on 9th of November 2021. There is emerging evidence to conclude that the Omicron variant spreading speed is much faster than the Delta and other previous variants, with the doubling time of only 2–3 days. On a global scale, the Omicron variant has been reported in 110 countries with the vast majority of confirmed cases coming from SA, the United Kingdom (UK), and the United States [4]. Sporadic cases were reported from some countries with no travel history in the US and Europe. A report from the UK Health Security Agency showed that up to 22 December 2021 there were more than 90,000 confirmed cases with 300 hospitalizations, and 24 deaths resulted from the Omicron. The probability of infection in a household when someone is infected with the Omicron was 2.09–3.2 times higher than that of the delta virus. The likelihood of intra-household indirect infection calculated by the available data for Omicron was 21.6% and 10.7% for Delta [8].

As it has been several weeks since the detection of Omicron, data about the clinical severity of this variant are still scarce and inadequate for an exact conclusion. However, although the Omicron has a higher risk of reinfection, a decrease of 29% in the hospitalization risk among Omicron-infected patients compared with other variants were witnessed in SA [9]. Several Omicron-infected cases only caused mild symptoms such as fatigue and itchy throat without any loss of taste and smell as well as any significant drop in oxygen concentration. However, these cases were associated with an outbreak from a university and mostly occurred in the young, who have a stronger immune system and a lower likelihood of severe clinical symptoms [9, 10].

Is fading immunity a characteristic of coronaviruses? In a study of the human 229E coronavirus, neutralizing antibodies blocked the 1984 variant but were less efficacious against the 1990s’ and 2010 variants [11]. The evolution and development of variants of COVID-19 may resemble the fast influenza A changes or the slow influenza B changes [12]. The former makes vaccine development more challenging and the second makes it more predictable [12].

**Fig. 1:**
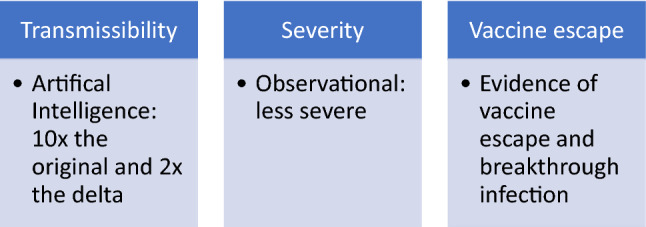
Areas of concerns for evolving SARS-CoV-2 variants

The answers to these three key questions about transmissibility, severity and vaccine escape are important to evaluate the significance of any variant (Fig. 1). It was suggested that the occurrence of specific mutations in the RBD and specifically of Y449S and N501Y result in decreased infectivity but leads to vaccine-resistance compared to the original SARS-CoV-2 variant [13]. The emergence of the Omicron variant had increased the debate about the need for COVID-19 vaccine booster dose as the current dosing regimen might not provide adequate antibody response to protect against this variant [14].

Using Artificial Intelligence (AI) modeling, it was calculated that the Omicron variant is 10 × and 2 × as contagious as the original SARS-CoV-2 variant and the Delta variant, respectively. This increase in infectivity is thought to be due to the N440K, T478K, and N501Y mutations [6]. The rapidity of transmission would result in a large number of infected individuals, and this would certainly result in a large number of severe cases even if the severity of the disease remains unchanged. The ability to evade the COVID-19 vaccine immunity and cause breakthrough infections due to the Omicron variant was also predicted to be higher than that of the Delta variant, due to K417N, E484A, and Y505H mutations [6]. The estimated re-infection hazard during the period from 01 to 27 November 2021 in SA (representing by Omicron variant) was 2.39 (95 CI 1.88–3.11) vs. the first wave of the epidemic [15]. The interpretation of laboratory data showing reduced neutralization from convalescent serum against the two important (Beta and Delta) variants when compared to the initial variant [16–18]. These laboratory data need to be correlated with clinical experience and rates of re-infection as well as rates of severe disease. In a recent data from SA, it was found that the risk of hospitalization is 29% lower than the risk in the first wave [19]. This might be explained by the findings that the Omicron variant is replicating in the human bronchus 70 times more than the delta variant and 10 times lower in the human lung tissue [19].

In a pre-print in vitro study, neutralizing antibodies against the Omicron variant were reduced at 11.4, 37.0, and 24.5 times for those who had two or three doses of the BNT162b2 vaccine and there was 20.0- and 22.7-fold reduction in those who had double mRNA1273 doses and were boosted with BNT162b2 vaccine [20]. On the other hand, there was no neutralizing effects from heterologously vaccinated ChAdOx1 and BNT162b2 [20]. An important finding was that BNT162b2- booster resulted in a significant increase of neutralizing antibodies with 27.1-fold reduction against Omicron [20].

Testing the effect of the inactivated whole-virion SARS-CoV-2 vaccine (CoronaVac) showed no detectable neutralizing antibodies against the Omicron variant and that the vaccinee with BNT162b2 had between 35.7 and 39.9-fold reduction in these antibodies compared to the ancestral SARS-CoV-2 virus [21]. Two other studies on the subject of neutralizing antibodies post BNT vaccination were conducted. A small number showed 29·eightfold reduction in neutralizing antibodies from homologous BNT recipients [22]. While the other study showed that there was a 22-fold reduction in neutralizing antibodies in both infected-vaccinated and vaccinated participants [23].

The diagnostic accuracy of routinely used PCR and antigen-based rapid diagnostic test (Ag-RDT) assays does not appear to be impacted by Omicron [5]. However, Omicron variant has a large number of mutations in the Spike protein (S). Genome sequencing data show that this new variant has a total of 50 mutations with 32 changes in the S protein [24], including Δ69–70. This deletion could lead to a drop-out of the S-gene assay in the TaqPath PCR test and thus this test could be used as a marker for the presence of the Omicron variant [25].

Although there were reports about the decreased efficacy of current vaccines against the Omicron variant, information to completely assess the efficacy of vaccines is still limited [20]. Scientists supposed that COVID-19 additional vaccines are still capable of protecting humans from variants, including Omicron. Rose et al. (2021) reported that an mRNA-1273 booster shot of vaccine could reduce the escape of Omicron variant from neutralizing antibodies [26]. Thus, governments are making efforts to give their residence booster shots of current vaccines. However, an additional shot of vaccine for each variant cannot be a long-term strategy. Since the existing mRNA and non-mRNA COVID-19 vaccines were based on the SARS-CoV-2 strain in Wuhan and lots of mutations were occurred in the Omicron as well as other variants, it may be time for developing a universal vaccine, which has entire-life effectiveness of protection [27–29]. Nevertheless, several mutations in the Omicron are overlapped with those in the previous variants (alpha, beta, gamma, and delta), a vaccine based on Omicron strain, maybe the mutated spike, can be expected to have a great efficacy of cross-protection [27]. In a preprint study, Kistler et al. (2021) suggested that the evolution of SARS-CoV-2 virus is much faster than influenza A as well as the seasonal coronavirus. The authors predicted that an update in the SARS-CoV-2 vaccines is required at least every 5 years [30].

With the rapid mutation and high transmissibility of SARS-CoV-2 virus, disease elimination is impossible, which means countries should instead develop their own strategies for control [31]. Firstly, expanding our current knowledge on the Omicron Variant COVID-19 via modern technology such as AI/machine learning for clarifying the pathogenesis and molecular mechanism are also urgently required, thus, comprehensive preventative methods and clinical management for Omicron-infected patients can be applied properly [31]. Secondly, implementation of booster shots of existing vaccines to minimize the detrimental impact of the Omicron on the community heath while waiting for new vaccines [27]. Finally, promoting the universal Coronavirus vaccines with much more efficacy to variants will be the most important strategy for the long-term battle against SARS-CoV-2 [29, 32, 33].

## Conclusion

The world is battling a new wave of the COVID-19 epidemic caused by the Omicron variant. It remains an open question regarding the origin, infectivity, virulence, and immunogenic escape of this variant. Large-scale coordinated studies are needed to evaluate the virulence of this variant and assess its impact on public health. Only then concerted efforts will be successful in implementing effective measures to reduce virus spread, morbidity and mortality. To succeed in controlling the global COVID-19 pandemic, we need to develop a safe and broadly protective coronavirus vaccines.

## Data Availability

Not applicable.

## Abbreviations

COVID-19: Coronavirus disease 2019
SARS-CoV-2: Severe acute respiratory syndrome coronavirus 2
WHO: World Health Organization
SA: South Africa
RBD: Receptor-binding domain
UK: United Kingdom
AI: Artificial intelligence

## References

[1] World Health Organization. WHO coronavirus (COVID-19) Dashboard n.d. https://covid19.who.int/ (Accessed April 21, 2021).

[2] World Health Organization (WHO). Tracking SARS-CoV-2 variants 2021. https://www.who.int/en/activities/tracking-SARS-CoV-2-variants/ (Accessed June 2, 2021).

[3] WHO. Classification of Omicron (B.1.1.529): SARS-CoV-2 variant of Concern 2021. https://www.who.int/news/item/26-11-2021-classification-of-omicron-(b.1.1.529)-sars-cov-2-variant-of-concern (Accessed December 10, 2021).

[4] Thakur V, Kanta Ratho R. OMICRON (B.1.1.529): a new SARS-CoV-2 variant of concern mounting worldwide fear. J Med Virol. 2021. 10.1002/JMV.27541. 10.1002/jmv.27541 34936120

[5] World Health Organization. Enhancing readiness for omicron (B.1.1.529): technical brief and priority actions for member states. World Heal Organ HQ 2021:1–8. https://www.who.int/publications/m/item/enhancing-readiness-for-omicron-(b.1.1.529)-technical-brief-and-priority-actions-for-member-states (Accessed December 25, 2021).

[6] Chen J, Wang R, Gilby NB, Wei G-W. Omicron (B.1.1.529): Infectivity, vaccine breakthrough, and antibody resistance. ArXiv 2021. 10.1021/acs.jcim.1c01451 PMC8751645 34989238

[7] Scott L, Hsiao N, Moyo S, Singh L, Tegally H, Dor G (2021). Track Omicron’s spread with molecular data. Science (80-).

[8] UK Health Security. COVID-19: Omicron daily overview - GOV.UK 2021. https://www.gov.uk/government/publications/covid-19-omicron-daily-overview (Accessed December 25, 2021).

[9] Ledford H (2021). How severe are omicron infections?. Nature.

[10] Kupferschmidt K, Vogel G (2021). How bad is Omicron? Some clues are emerging. Science.

[11] Eguia RT, Crawford KHD, Stevens-Ayers T, Kelnhofer-Millevolte L, Greninger AL, Englund JA (2021). A human coronavirus evolves antigenically to escape antibody immunity. PLoS Pathog.

[12] Callaway E (2021). Beyond omicron: what’s next for COVID’s viral evolution. Nature.

[13] Wang R, Chen J, Wei G-W (2021). Mechanisms of SARS-CoV-2 evolution revealing vaccine-resistant mutations in Europe and America. J Phys Chem Lett.

[14] Graham F (2021). Daily briefing: omicron—what scientists know so far. Nature.

[15] Pulliam JRC, van Schalkwyk C, Govender N, von Gottberg A, Cohen C, Groome MJ (2021). Increased risk of SARS-CoV-2 reinfection associated with emergence of the Omicron variant in South Africa. MedRxiv.

[16] Liu C, Ginn HM, Dejnirattisai W, Supasa P, Wang B, Tuekprakhon A (2021). Reduced neutralization of SARS-CoV-2 B.1.617 by vaccine and convalescent serum. Cell.

[17] Wibmer CK, Ayres F, Hermanus T, Madzivhandila M, Kgagudi P, Oosthuysen B (2021). SARS-CoV-2 501Y.V2 escapes neutralization by South African COVID-19 donor plasma. Nat Med.

[18] Planas D, Veyer D, Baidaliuk A, Staropoli I, Guivel-Benhassine F, Rajah MM (2021). Reduced sensitivity of SARS-CoV-2 variant delta to antibody neutralization. Nature.

[19] Dyer O (2021). Covid-19: omicron is causing more infections but fewer hospital admissions than delta. South African data show Bmj.

[20] Wilhelm A, Widera M, Grikscheit K, Toptan T, Schenk B, Pallas C (2021). Reduced Neutralization of SARS-CoV-2 Omicron Variant by Vaccine Sera and monoclonal antibodies. MedRxiv.

[21] Lu L, Mok BW-Y, Chen L-L, Chan JM-C, Tsang OT-Y, Lam BH-S (2021). Neutralization of SARS-CoV-2 Omicron variant by sera from BNT162b2 or Coronavac vaccine recipients. Clin Infect Dis.

[22] Dejnirattisai W, Shaw RH, Supasa P, Liu C, Stuart AS, Pollard AJ (2021). Reduced neutralisation of SARS-CoV-2 omicron B.1.1.529 variant by post-immunisation serum. Lancet.

[23] Cele S, Jackson L, Khoury DS, Khan K, Moyo-Gwete T, Tegally H (2021). SARS-CoV-2 Omicron has extensive but incomplete escape of Pfizer BNT162b2 elicited neutralization and requires ACE2 for infection. MedRxiv Preprin.

[24] Quarleri J, Galvan V, Delpino MV (2021). Omicron variant of the SARS-CoV-2: a quest to define the consequences of its high mutational load. GeroScience.

[25] Ferré VM, Peiffer-Smadja N, Visseaux B, Descamps D, Ghosn J, Charpentier C (2021). Omicron SARS-CoV-2 variant: what we know and what we don’t. Anaesth Crit Care Pain Med.

[26] Doria-Rose NA, Shen X, Schmidt SD, O’Dell S, McDanal C, Feng W (2021). Booster of mRNA-1273 vaccine reduces SARS-CoV-2 omicron escape from neutralizing antibodies. MedRxiv Prepr Serv Heal Sci.

[27] Burki TK (2021). Omicron variant and booster COVID-19 vaccines. Lancet Respir Med.

[28] Li X (2021). Omicron: call for updated vaccines. J Med Virol.

[29] Cohen J (2021). Omicron sparks a vaccine strategy debateCurrent boosters, variant-specific shots, and universal coronavirus vaccines all have fans. Science (80-).

[30] Kistler KE, Huddleston J, Bedford T (2021). Rapid and parallel adaptive mutations in spike S1 drive clade success in SARS-CoV-2. BioRxiv.

[31] Wang X, Powell CA (2021). How to translate the knowledge of COVID-19 into the prevention of Omicron variants. Clin Transl Med.

[32] He X, Hong W, Pan X, Lu G, Wei X (2021). SARS-CoV-2 omicron variant: characteristics and prevention. MedComm.

[33] Morens DM, Taubenberger JK, Fauci AS (2021). Universal coronavirus vaccines—an urgent need. N Engl J Med.

